# Capacity of Autonomous Sensory Meridian Response on the Reduction of Mental Stress

**DOI:** 10.3390/ijerph192114577

**Published:** 2022-11-06

**Authors:** Keiichiro Inagaki, Yoshiyuki Ohta

**Affiliations:** College of Engineering, Chubu University, 1200 Matsumoto, Kasugai 487-8501, Aichi, Japan

**Keywords:** EEG, ASMR, Fourier spectra, mental workload

## Abstract

In a social environment, various types of stress can be overwhelming. Humans frequently encounter these stressful situations in social life. Stress is divided into physical stress and mental stress; the latter is induced by heavy mental workloads and has become a huge social problem, leading to mental disorders and possibly suicide in the worst scenario. Investigations into monitoring mental stress and reducing stressful conditions are, therefore, important for its prevention. In the present study, we focused on autonomous sensory meridian response (ASMR) sound, which is known to improve the human mental condition through its comforting and relaxing effects. We investigated the effect of ASMR on the mental workload induced by mental tasks by the evaluation of EEG activation patterns in normal subjects. Our results showed a significant decrease in alpha-band activity and a significant increase in gamma (high beta)-band activity under the induction of mental workload by mental tasks compared to the resting condition. When applying ASMR sound, alpha- and gamma-band activity under the induction of mental workload by mental tasks was restored to the level of the resting condition. In conclusion, these results indicate that ASMR sound reduces the mental stress induced by mental workload.

## 1. Introduction

In social life, humans frequently encounter a wide variety of stressful conditions. Most humans living in society experience huge stress and anxiety in the working environment [1], indicating that working stress is common in the current social system. It is known that stress and anxiety, especially mentally related, are deeply related to mental disease and disorders [1,2,3]; namely mental stress directly affects cardiovascular disease, speech distinction, cognitive dysfunction, etc. [3,4,5]. Mental stress is caused by the huge workload induced by a variety of working tasks [1]; therefore, it has become one of the crucial social problems. To avoid this issue, it is important to study and develop monitoring, evaluation, and reduction methods of mental workload related to mental stress.

Several lines of investigation have been performed to understand the relationship between mental stress and biological information, in which mental stress was evoked by several types of mental tasks: maze, mental arithmetic, math calculation, form perception, geometric construction, etc. [6,7,8,9,10]. Several types of biometric information, cortisol level, heart rate activity, electrodermal activity, thermography, brain activity measured by fNIRS, fMRI, PET, and electroencephalogram (EEG) have been utilized to evaluate the effects of mental stress [11,12,13,14,15,16,17,18,19,20,21,22,23,24,25]. Among these, EEG has many advantages in terms of cost, high temporal resolution, and ease of use [17]. The EEG activation pattern reflecting mental stress varies with the working environment [26,27,28,29,30,31]. For instance, classical or pleasant music activates more alpha and theta-band activity and suppresses more beta-band activity, whereas up-tempo music evokes the opposite activation patterns. These results suggest that EEG also has the advantage of evaluating the multimodal effects such as instrumentation on the mental stress induced experimentally using mental tasks.

In the present work, we focused on the effect of ASMR (autonomous sensory meridian response). ASMR is a tingling sensation often induced by certain auditory or visual stimuli [32]. It is reported that this sensation causes relaxation and content [33], leading someone to reduce human mental stress and its related disorders [32,34,35,36]. Moreover, the ASMR changes functional connectivity along with feeling a tingling sensation [37,38], eliciting frontal-lobe alpha wave activity which is related to the attentional and sensorimotor phenomenology [39], activating in regions associated with both reward and emotional arousal [40]. The effect of ASMR on brain activity has been gradually uncovered; however, the effect of ASMR on human brain activity under the induction of mental stress, which is mostly caused by an induction of mental workload while performing mental tasks, remains a mystery. In this study, we investigate human brain activity (EEG) under the induction of mental workload during the performance of mental tasks along with an application of ASMR. We evaluate changes in spectral response and evaluate the relationship between these changes and mental workload assessed by NASA-TLX that lead to mental stress to investigate the recovering effect of ASMR on brain activity during mental workload.

## 2. Materials and Methods

### 2.1. Subjects

EEGs were recorded from 12 healthy male subjects aged 19 to 24 years (21.33 ± 1.49) without health or neurological problems and their antecedents. All subjects also passed audiometry. Before the experiments, the experimental outline was explained to all subjects to obtain informed consent.

### 2.2. Experimental Setup and Tasks

**Figure 1:**
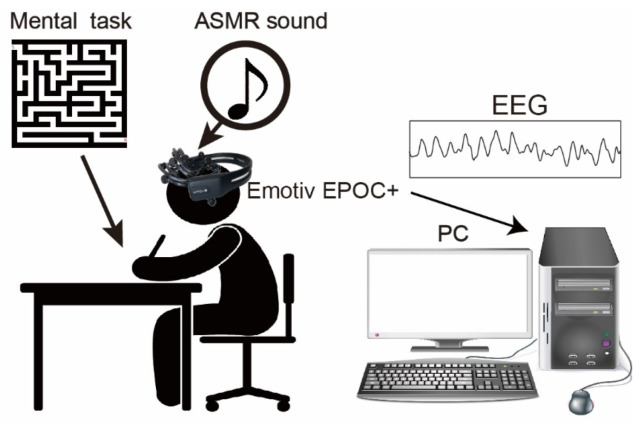
The experimental setup and mental task. Subjects sat on the chair and performed the mental task. The 40 × 40 size mazes printed on A4 paper were employed as our mental task, and the subject continuously solved them for 3 min. An Emotiv EPOC+ (14 channels) was utilized to measure human EEG. In the experiment, the ASMR sound was given by a headphone.

Figure 1 summarizes the experimental setup for measuring EEGs during mental tasks. A headphone with individually tuned volume at the range of less than 80dB was utilized to hear the ASMR sound and shut out any background sound in the experimental room. The ASMR sound utilized in the experiment was classified into 6 types based on onomatopes (kata-kata (typing keyboard, https://www.youtube.com/watch?v=8L1E9O9LgKo (accessed on 19 October 2022)), saku-saku (eating crunchy food, https://www.youtube.com/watch?v=CpbRfUMehmg (accessed on 19 October 2022)), syuwa-syuwa (fizzy sound of sparkling water, https://www.youtube.com/watch?v=a3h-K5cBRiQ (accessed on 19 October 2022)), gori-gori (sound of earpick, https://www.youtube.com/watch?v=2gYEKqOU6yk (accessed on 19 October 2022)), zowa-zowa(scratching a sponge, https://www.youtube.com/watch?v=u1zELxKVaxc (accessed on 19 October 2022)), shyaki-shyaki (sound of chopping leafy vegetable, https://www.youtube.com/watch?v=RfMz0udnmcY (accessed on 19 October 2022)). The ASMR were obtained from YouTube, and only sound is extracted. The duration of the ASMR sound was also adjusted to the duration of the experimental condition described below. In the experiment, a different pattern of 40 × 40 sizes of mazes created web apps (http://www.kiy.jp/~yoka/gameland/labyrinth/labyrinth_JS.cgi (accessed on 19 October 2022)) and printed on individual paper was utilized as mental tasks [8]. It is reported that the mental stress/condition could be changed for continuously solving the mazes for 2 min [8], therefore, we set the duration for continuously solving the mazes to 3 min.

### 2.3. Experimental Protocol

**Figure 2:**
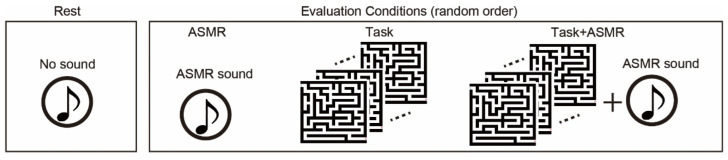
Experimental condition for evaluation of the effect of ASMR on the mental stress. We use resting condition(rest), and three types of evaluation condition: sole application of ASMR sound (ASMR), sole application of maze tasks (Task), and simultaneous application of maze task and ASMR sound (Task + ASMR). NASA-TLX is performed after Task and Task + ASMR. Duration of all of condition is 3 min. The evaluation conditions are assessed in random order.

The subjects listened to 6 types of ASMR sound and chose the most comfortable one preceding the EEG measurement to be utilized as external sound input due to that the type of sound makes a difference individually in whether ASMR can be felt [36]. Figure 2 summarized four experimental conditions: resting and 3 different evaluation conditions. In the resting condition, subjects keep relaxing with eyes open (Rest). In the evaluation conditions, we used to follow three types of the condition: sole application of ASMR sound (ASMR), sole application of maze tasks (Task), and simultaneous application of maze tasks and AMSR sound (Task + ASMR). Duration of all conditions is 3 min. In the Task condition, subjects solve the maze tasks as much as they can during 3 min. In the Task + ASMR condition, subjects also solve the maze tasks as much as they can during 3 min with listening to the ASMR sound. In the ASMR condition, subjects solely listened to the ASMR sound for 3 min with eyes open. In the experiment, we first measured the EEG under the resting condition. Then, these evaluation conditions were presented in random order and measured EEG during those conditions. Between conditions, we gave a 1-min rest time for subjects.

To evaluate their mental workload, we asked the subject to answer the NASA-Task Load Index (NASA-TLX, see below) [41] after that subjects performed Task or Task + ASMR condition. The whole experiment was conducted between 2 p.m. and 5 p.m. to avoid the effects of the circadian rhythm. The arousal conditions of all subjects were checked before the experiment using the Karolinska Sleepiness Scale [42] translated to Japanese, and subjects who reported more than level 3 which is the criteria of drowsy did not attend this experiment. The Ethics Committee of Chubu University approved the experimental protocol (#20220007). We performed all methods in accordance with the Declaration of Helsinki.

### 2.4. Measurement of EEG

EEGs of all subjects were measured using an Emotiv EPOC+ (Emotive, CA, US) with a 128-Hz sampling frequency. To record EEG data, electrodes (14 channels) were placed according to the international 10–20 electrode position system [43]. During the placement of the electrodes, the scalp of the subject was cleaned using alcohol to avoid EEG signal deterioration due to scalp oil. In this study, we selected O1/O2 for the measurement of EEG and its analysis because our task-related vision and visual task-related mental stress were observed in the occipital area [22,44,45,46,47], and there was less noise from eye blinking and eye movements in this study.

### 2.5. Analysis

Fast Fourier transform (FFT) was used to obtain the power frequency spectrum. At first, we checked the huge trend and quick phasic (spiky) noise in EEG signals. The EEG data that do not involve those artifacts were utilized in the analysis. In the application of FFT, the DC component of EEG data was removed after the application of a band-pass filter (3–50 Hz). FFT was applied every second with a Hanning filter for the whole data. Finally, the obtained power frequency spectrum was averaged. The determined power spectrum was converted to a probability distribution *P*(*k*) as follows:

(1) $$P\left(k\right)=\frac{S\left(k\right)}{\sum_{k=1}^{f_{n}}S\left(k\right)}$$

where *S*(*k*) is the power spectrum at the frequency *k*, and *f_n_* is the Nyquist frequency (64 Hz). We divided the spectral responses for the alpha-band (8–13 Hz) and gamma-band including the high beta-band (25–40 Hz) and calculated an average for each frequency band. Statistical significance was evaluated using the Wilcoxon ranked sum test with adjusting Benjamini-Hochberg methods (FDR < 0.05) [48] due to multiple comparisons. We also validated the number of subjects using G*power [49] (type I error < 0.05 and type II error = 0.8), and confirmed that all pair showing significance meets the estimated sample size, in which the effect size was also calculated by Cohen’s *d* [50].

### 2.6. Evaluation of Mental Workload

The mental workload caused by the mental tasks was evaluated by means of NASA-TLX [41]. NASA-TLX is the subjective measurement of the workload and can estimate mental workload by considering mental demand (MD), physical demand (PD), temporal demand (TD), own performance (OP), effort (EF), and frustration (FR). In our experiment, the score of each index was evaluated by a point ranging from 0 to 20, and the volume of mental workload was high when these scores became high. We asked subjects to report NASA-TLX at the end of performing the mental task with/without applying ASMR sound. The averaged workload (*S_wwl_*) was calculated as follows:

(2) $$S_{wwl}=\left(S_{md}+S_{pd}+S_{td}+S_{op}+S_{ef}+S_{fr}\right)/6$$

*S_md_*, *S_pd_*, *S_op_*, *S_ef_*, and *S_fr_* are the score of each NASA-TLX index.

To investigate the relationship between changes in OP and task performance in the continuously solving mazes without the ASMR sound and with the ASMR sound, we checked the number of completed maze tasks.

## 3. Results

**Figure 3:**
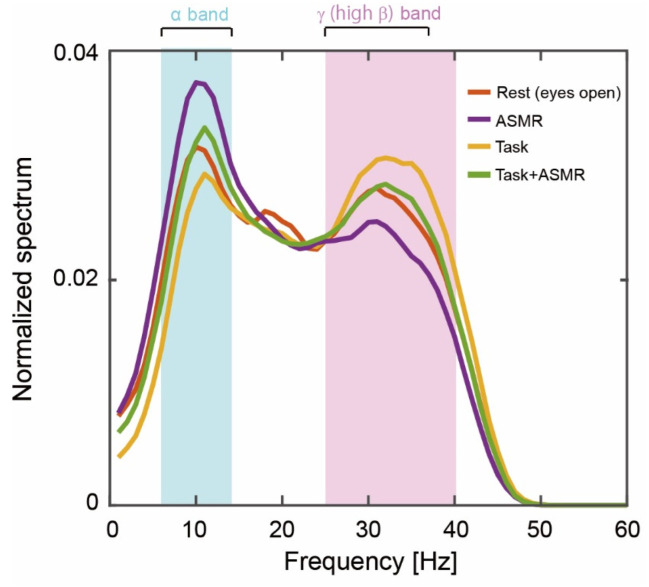
Grand averages of the power spectrum during our experiments. The solid orange, purple, yellow, and green lines indicate the resting condition with open eyes, listening to ASMR sound with open eyes, performing mental tasks without any ASMR, and performing the mental task while listening to ASMR sound, respectively. Pale blue and red areas show alpha-band (6–13 Hz) and gamma (high beta, 25–38 Hz)-band activity.

Figure 3 summarizes the grand averages of spectral responses calculated from 12 subjects at resting condition (Rest), resting condition with ASMR sound (ASMR), and during the mental tasks with and without ASMR sound (Task and Task + ASMR). For the alpha band response (pale blue area in Figure 3), the largest activation pattern was observed in the resting condition, and it was further elevated by the application of ASMR. The smallest activation pattern was observed during the performance of mental tasks without ASMR sound. This depressed spectral response was almost recovered to resting condition by the application of ASMR during a mental task. For the gamma band including high beta band response, the opposite activation pattern was observed for those experimental conditions (See the pale red area in Figure 3).

**Figure 4:**
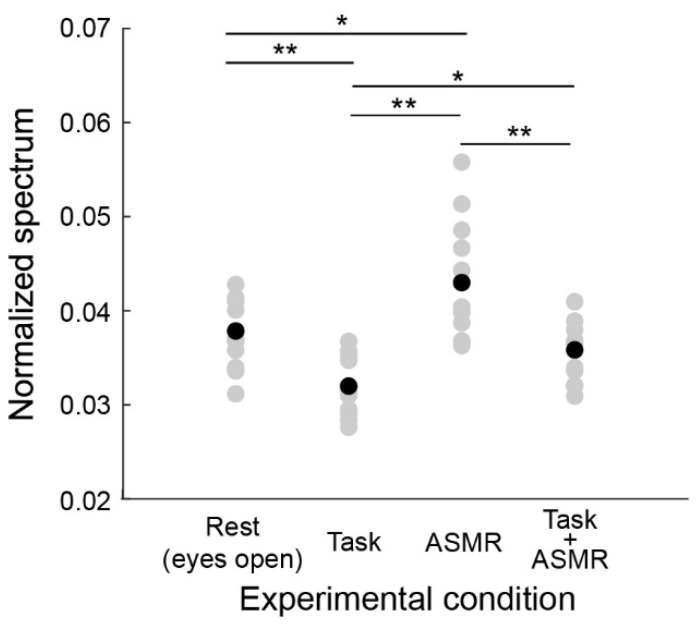
Average power spectral response for alpha band activity in each experimental condition. Gray and black-filled circles are individual and averaged responses, respectively. Asterisks * and ** indicate *p* < 0.05 and *p* < 0.01 as evaluated by Wilcoxon ranked sum test with adjusting by Benjamini-Hochberg methods.

Figure 4 summarizes the alpha band EEG activation pattern during the resting condition and the evaluation condition. The average and standard deviation for each condition are 0.0379 ± 0.0029 (Rest), 0.0315 ± 0.0031 (Task), ASMR (0.0432 ± 0.0058), and Task + ASMR (0.0354 ± 0.0034), respectively. In these results, we statistically compared the alpha band activity for each condition using Wilcoxon ranked sum test with adjusting by Benjamini-Hochberg methods (FDR < 0.05) due to multiple comparisons and summarized those in Table 1. During the performance of the mental task, significantly lower alpha-band activity was observed in all subjects, but it was higher in the resting condition (*p* < 0.01 and Cohen’s *d* = 1.34 in Rest vs. Task). However, when applying ASMR sounds during the mental task, the level of alpha-band activity was almost restored to the resting condition (*p* < 0.05 and Cohen’s *d* = 1.16 in Task vs. Task + ASMR). There was no significant difference between the resting condition and mental task when ASMR sounds were applied. These results seem to show that ASMR reduced the mental stress induced by mental workload. When solely applying ASMR during the resting condition, a significant increase in alpha-band activity was observed compared to the resting condition without ASMR sounds, which also implies the contribution of ASMR to relaxation (*p* < 0.05 and Cohen’s *d* = 0.88, Rest vs. ASMR).

**Figure 5:**
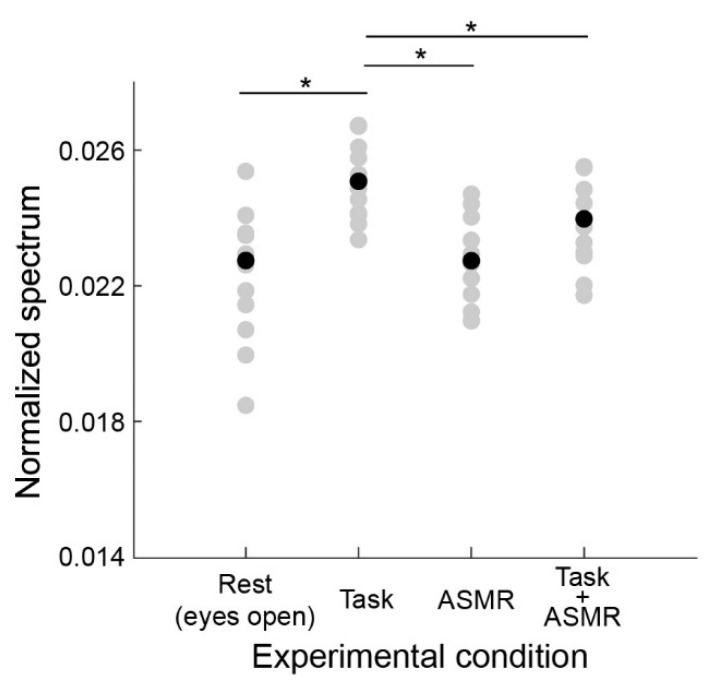
Average power spectral response for gamma band activity in each experimental condition. Gray and black-filled circles are individual and averaged responses, respectively. Asterisks * indicates *p* < 0.05 as evaluated by Wilcoxon ranked sum test with adjusting by Benjamini-Hochberg methods. Depicted in the same format as Figure 4.

Figure 5 summarizes the gamma-band EEG activation pattern during the resting condition and the mental task. The average and standard deviation of gamma-band spectral response for each condition are 0.0224 ± 0.0020 (Rest), 0.0248 ± 0.0011 (Task), ASMR (0.0229 ± 0.0011), and Task + ASMR (0.0234 ± 0.0013), respectively. In these results, we statistically compared the gamma band activity for each condition using Wilcoxon ranked sum test with adjusting by Benjamini-Hochberg methods (FDR < 0.05) due to multiple comparisons and summarized those in Table 2. During the performance of the mental task, a significant gamma-band activity was observed in all subjects (*p* < 0.05 and Cohen’s *d* = 1.05 in Rest vs. Task). However, when applying ASMR sound during the mental task, the gamma-band activity was restored to a level slightly higher than the resting condition (*p* < 0.05 and Cohen’s *d* = 1.08 in Task vs. Task + ASMR). Here, we confirmed no significant difference between the resting condition and mental task with ASMR. Similarly, significant differences were not observed between the resting condition and the resting condition with ASMR.

**Figure 6:**
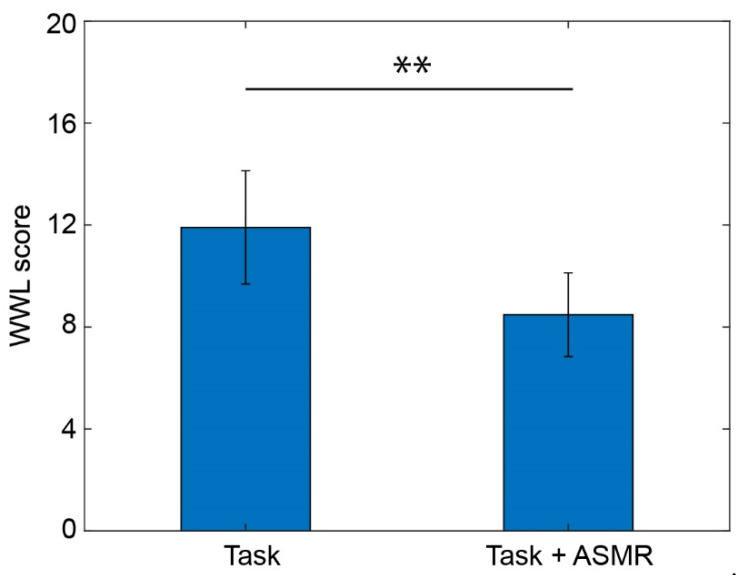
Averaged workload calculated from NASA-TLX while performing the mental task without (**left**) and with (**right**) application of ASMR sound. Asterisks ** indicate *p* < 0.01 as evaluated by Wilcoxon ranked sum test.

The effect of mental tasks and the ASMR sound were evaluated by an index for mental workload called NASA-TLX. Figure 6 illustrates the mental workload evaluated using the averaged workload of NASA-TLX during the performance of mental tasks with/without ASMR sound. The average workload during the mental task was significantly decreased by the application of ASMR sound (*p* < 0.01 and Cohen’s *d* = 2.03).

**Figure 7:**
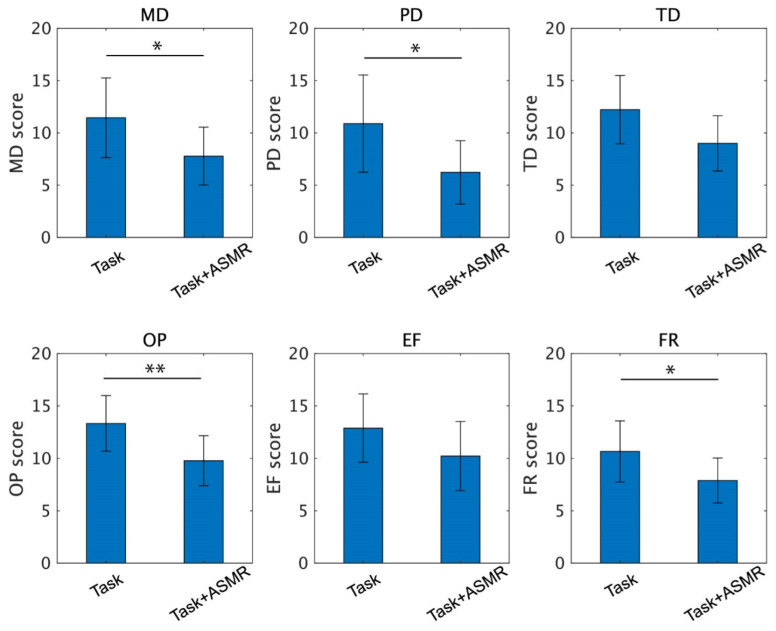
Individual scores of the NASA-TLX for the performance of the mental task without (**left**) and with (**right**) application of ASMR sound. Asterisks * and ** indicate *p* < 0.05 and *p* < 0.01 as evaluated by Wilcoxon ranked sum test.

Figure 7 summarizes each index of NASA-TLX taken from 12 subjects. From these indexes, the ASMR sound significantly depressed the scores for mental demand (MD, *p*<0.05 and Cohen’s *d* = 1.35), physical demand (PD, *p* < 0.05 and Cohen’s *d* = 1.32), frustration (FR, *p* < 0.05 Cohen’s *d* = 1.67), and own performance (OP, *p* < 0.01 and Cohen’s *d* = 1.21). The indexes for temporal demand (TD) and effort (EF) were also decreased by the application of ASMR sound during the performance of the mental task, but the differences were not significant (*p* = 0.051 for TD and *p* = 0.119 for EF). The number of complete maze tasks in the condition with and without the ASMR is 1.83 ± 0.55 and 2.66 ± 1.02, respectively, and significance evaluated by Wilcoxon ranked sum test is observed (*p* < 0.05 and Cohen’s *d* = 0.79).

## 4. Discussion

ASMR sound or video causes a peculiar tingling sensation in peoples’ heads, and most people feel this sensation relaxing [32,33]. In the present study, we investigated the effect of the ASMR sound during the performance of mental tasks by measuring EEG. Both depressed alpha-band and boosted gamma-band activities caused by mental workload induced by performing mental tasks were recovered to the level of the resting condition by the ASMR. Furthermore, the alpha-band activity in subjects listening to ASMR was increased from the activity during the resting condition. These results indicated that the ASMR has two roles: one is relaxation as reported in previous studies as an elevation of alpha-band EEG activity, and the other is the capacity of recovering mentally stressful conditions caused by the induction of mental workload using mental tasks. This recovering effect of ASMR on brain activity during mental workload was revealed by our study for the first time and implies the possibility that ASMR might be used as a key sensation to suppress mental stress induced by mental workload and to prevent especially work-related mental disorders. Note that the fact that the effects of ASMR are individually dependent [36]. Namely, the type of sound makes a difference individually in whether ASMR can be felt; therefore, it is important to select the most effective type of ASMR sound for use to improve mental conditions.

In our results, the alpha-band activity was depressed during the mental task, and the gamma-band activity was elevated. These changes indicated that the subjects performing our mental tasks experienced similar mental stress as reported in previous research [26,27,28,29,30,31]. However, ASMR sound solely elevated alpha-band activity in subjects performing no tasks. Those changes in EEG activation patterns are related to that ASMR caused the subjects to relax, as shown in previous studies [32,34,35,36]. Under the occurrence of those changes of EEG response in the alpha-band and gamma-band including high beta-band, the mental workload induced by our mental task was evaluated by NASA-TLX [41]. Although the mental workload was elevated by continuously performing the mental task without any application of acoustic input to the subjects, the elevated workload was significantly recovered by applying ASMR sounds during the performance of the mental task. Individual components of the NASA-TLX, the MD, PD, OP, and FR, were significantly reduced by the ASMR even though subjects continued to perform the mental task. These changes support the idea that ASMR sound has the capacity to recover mentally stressful conditions. Several studies have reported that the sound environment was effective in the performance of a task [51,52,53,54,55,56,57,58]. Our results also show that the OP score was reduced by the ASMR by significantly increasing task performance evaluated by the number of completed maze tasks. This result finding by subjective index NASA-TLX implied the possibility of ASMR to improve task performance, and we further investigated this possibility by considering an objective index for task performance in the future study.

Human emotions track the changes in the acoustic/music environment. For instance, sedative music is highly correlated with calmness, relaxation, and tenderness, whereas stimulative music is correlated with tension, anger, and boldness [55]. Moreover, certain types of music reduce anxiety or facilitate the performance of a task [51,52,53,54,55,56,57,58,59,60,61]. In the case of evaluation of mental stress, it is also known that classic or pleasant music reduces the mental stress induced by the task [53,54,55,59,60,61]. In our results, the ASMR sound has recovering effect on brain activity related to mental stress induced by mental workload, indicating ASMR might have a similar effect such as relaxation reported in the sedative, classic or pleasant even if the type of music is different, and this similarity on the effect of mental stress need to be studied in the future study.

In the case of the ASMR used in this study, it is also controversial whether the effect of ASMR on the reduction of mental conditions might be a “placebo” [62]. Our current results might answer this question because the ASMR reverted the brain activity induced by the mental workload to the resting level. However, we did not compare the effects of sounds among other varieties such as classic music, healing music, popular music, etc. Therefore, we will do this comparison in a future study.

Although the recovery of mentally stressful conditions by ASMR sound was observed in our EEG study, we could not uncover the changes in neuronal function or neuronal mechanisms by merely analyzing the spectral response of a particular electrode position. It was reported using resting-state fMRI that functional connectivity is reduced by the ASMR experience relative to subjects who did not experience ASMR [37]. In addition, ASMR elevates the functional connectivity related to default mode networks [63,64]. Therefore, future studies should use a functional connectivity analysis to understand the relationship between changes in neuronal signal connections and the recovery of mental stress by ASMR sound.

Finally, the possibility of the ASMR on the reduction of mental stress induced by the mental workload is implied by our study. However, this finding is yielded by this experimentally limited condition, for instance, the use of subjects free of neurological and psychiatric conditions and non-ecological mental tasks which are not experienced in real life. The personal and clinical use of the ASMR might reduce mental stress and its related disorder and suicide in the worst case. Therefore, further investigation of the effect of ASMR on the reduction of mental condition in the patient with mental diseases with considering the way of utilization in the future study.

## 5. Conclusions

We investigated the effects of ASMR on the activity pattern of human EEG during the performing of a mental task. We confirmed a significant change in both alpha-band and gamma-band activities between the resting condition and the condition of performing mental tasks. Furthermore, no significant difference in activated EEG pattern was found between performing the mental tasks with ASMR and resting condition. These results showed that ASMR sound recovered the EEG activation pattern even during the performing of mental tasks, and conclude that ASMR might have the capacity to reduce mental stress caused by mental workload. Finally, these findings might be one clue that the utilization of the ASMR has the possibility to prevent mental disorders and related suicides caused by mental stress, and this possibility will need to be clinically assessed in the future study.

## Figures and Tables

**Table 1:** Statistics results for alpha-band avidity of with adjusting by Benjamini-Hochberg methods (FDR < 0.05).

Condition	*p*-Value	Rank	Adjusted-*p*	BH Significance	Cohen’s *d*
**Task-ASMR**	0.0000469	1	0.0002814	Yes	1.64
**Rest-Task**	0.00047768	2	0.00143304	Yes	1.34
**ASMR-Task+ASMR**	0.0043	3	0.0086	Yes	1.08
**Task-Task+ASMR**	0.0166	4	0.0249	Yes	1.16
**Rest-ASMR**	0.0411	5	0.04932	Yes	0.88
**Rest-Task+ASMR**	0.126	6	0.126	No	0.56

**Table 2:** Statistics results for gamma-band avidity of with adjusting by Benjamini-Hochberg methods (FDR < 0.05).

Condition	*p*-Value	Rank	Adjusted-*p*	BH Significance	Cohen’s *d*
**Task-ASMR**	0.0024	1	0.0144	Yes	1.84
**Rest-Task**	0.0035	2	0.0105	Yes	1.05
**Task-Task+ASMR**	0.0226	3	0.0452	Yes	1.08
**Rest-Task+ASMR**	0.2602	4	0.3903	No	0.34
**ASMR-Task+ASMR**	0.3708	5	0.4449	No	0.22
**Rest-ASMR**	0.7075	6	0.7075	No	0.30

## Data Availability

Datasets generated during the current study are available from the corresponding author on reasonable request.
